# Comparing Nuclear Reaction Coordinates Driving Photoinduced Proton-Coupled Electron and Energy Transfer

**DOI:** 10.1021/jacs.6c15580

**Published:** 2026-10-26

**Authors:** Andreas Ghosh, Kai Cui, D. Vale Cofer-Shabica, Joseph E. Subotnik, Sharon Hammes-Schiffer

**Affiliations:** Department of Chemistry, 6740 Princeton University, Princeton, New Jersey 08544, United States

## Abstract

Proton transfer is coupled to electron transfer or electronic excitation energy transfer in proton-coupled electron transfer (PCET) and proton-coupled energy transfer (PCEnT) reactions, respectively. Although photoinduced PCET and PCEnT have been studied extensively in anthracene–phenol–pyridine molecular triads, the nuclear reaction coordinates for these two pathways are still unknown. Herein, we identify the nuclear reaction coordinates for PCET and PCEnT in the triads by sampling the seam space between the relevant intersecting diabatic states. The reaction coordinate has contributions mainly from anthracene and phenol for PCET but involves the entire triad, including pyridine, for PCEnT. Both reaction coordinates involve motions related to contraction of the phenol C–O bond as well as motions within the anthracene rings that differ for PCET and PCEnT due to different charge distributions. The proton acceptor motion contributes more to the PCEnT than the PCET reaction coordinate. Both PCET and PCEnT reactions are concerted in that there is no stable intermediate, but they are both most likely asynchronous. Our analysis also suggests that PCET proceeds through a conical intersection in its minimum energy pathway, whereas PCEnT moves through an avoided crossing. The quantum mechanical treatment of the transferring proton within a vibronically nonadiabatic framework suggests that the kinetic isotope effect may be greater for PCEnT than for PCET, although more extensive calculations are required for confirmation. Understanding the fundamental similarities and differences between PCET and PCEnT mechanisms will help guide the design of proton-coupled systems that favor either charge transfer or energy transfer.

## Graphical abstract

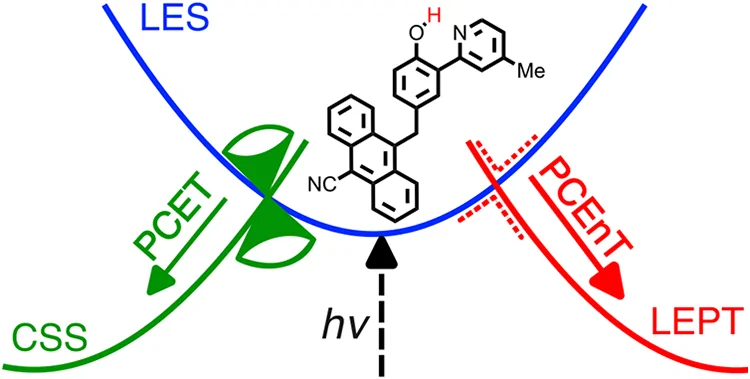

## 1 Introduction

Electron transfer (ET) and electronic excitation energy transfer (EnT) are essential for a wide range of chemical and biological processes. For example, both processes feature prominently in photosynthesis: solar energy is harvested through EnT in Photosystem II,^1–7^ and an electron–hole pair is generated through ET in Photosystem I.^8–12^ Often these elementary steps are coupled to proton transfer to avoid high-energy intermediates and reach more thermodynamically stable products. In the case of ET, this phenomenon is known as proton-coupled electron transfer (PCET), which occurs in many biological^13–16^ and electrochemical^17–20^ systems beyond photosynthesis. In the case of EnT, this phenomenon is known as proton-coupled energy transfer (PCEnT), which was discovered more recently.^21–26^

Because of its ubiquity in biological environments, many biomimetic systems have been constructed to elucidate the fundamental nature of PCET. One such example is the anthracene–phenol–pyridine triads (henceforth referred to as “triads”) shown in Figure 1. After photoexcitation of the anthracene from the ground state to the locally excited state (LES), an electron from the phenol transfers to the anthracene, while a proton transfers from the phenol to the pyridine. This PCET reaction results in the long-lived charge separated state (CSS). Subsequently, the CSS relaxes back down to the ground state through a charge recombination PCET reaction, which was shown to exhibit Marcus inverted-region behavior.^27^ Figure 2 shows the natural transition orbitals^28,29^ associated with the LES and CSS in triad **1**. This PCET reaction has been studied with vibronically nonadiabatic PCET theory.^27,30–32^

**Figure 1:**
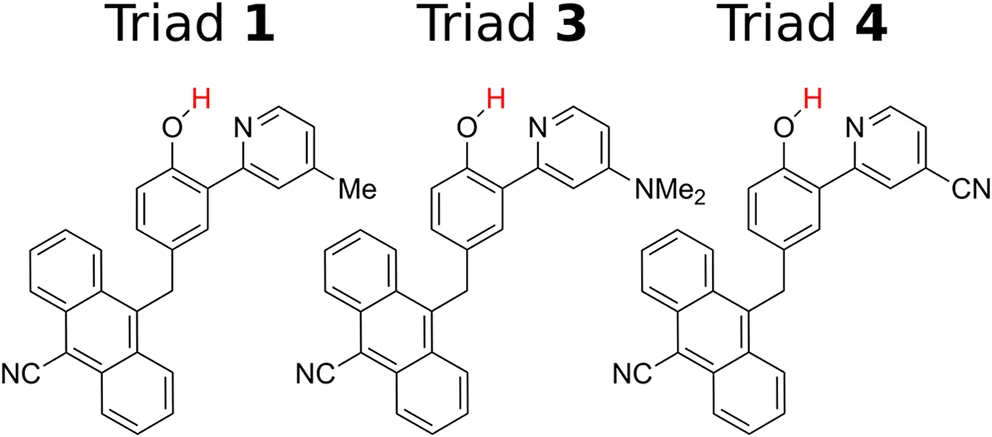
Anthracene−phenol−pyridine triads examined in this study, with the transferring proton highlighted in red. Note that the only difference between the triads is the group on the pyridine. We studied both PCET and PCEnT in triad **1**, PCET in triad **3**, and PCEnT in triad **4**.

**Figure 2:**
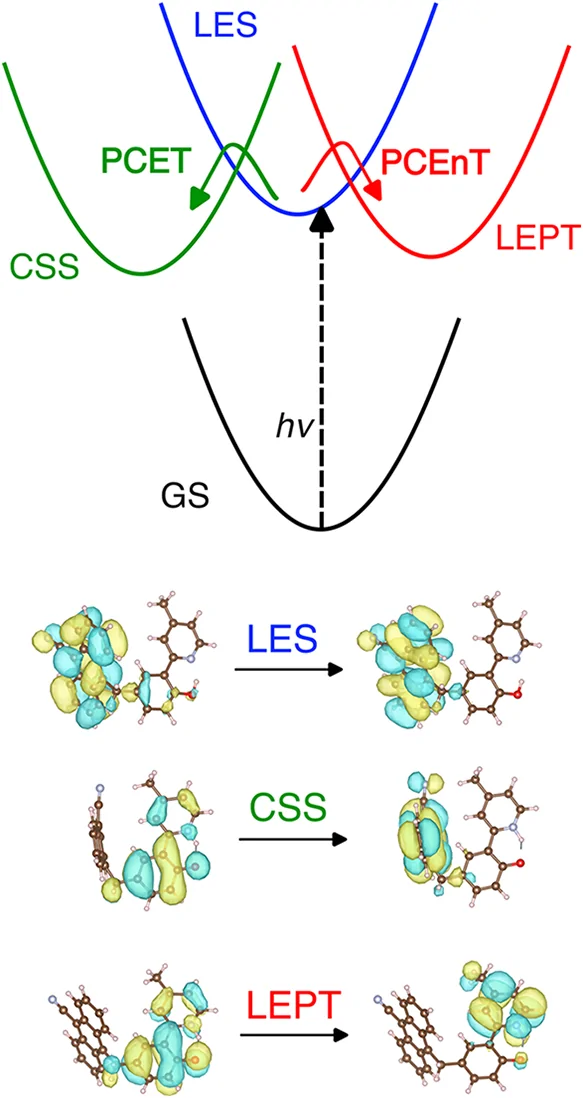
Top: schematic of the ground state and relevant excited states. The LES–CSS transition corresponds to PCET, and the LES–LEPT transition corresponds to PCEnT. Bottom: dominant natural transition orbitals for the LES, CSS, and LEPT state of triad **1** obtained from TDDFT calculations. These orbitals show the occupied to virtual transitions that characterize these excited states. The isosurface value is 0.02 Å^–3/2^.

Theoretical studies predicted that such a PCET reaction is not the only available reaction pathway for the triads. Multireference calculations identified another type of excited state, the local electron–proton transfer (LEPT) state,^33^ which could be brought lower in energy than the CSS with a more electron-withdrawing group on the pyridine. Subsequently, nonequilibrium first-principles molecular dynamics simulations on a time-dependent density functional theory (TDDFT) potential energy surface showed that the LEPT state could be accessed directly from the LES, providing an alternative relaxation pathway for certain triads.^34^ The natural transition orbitals for the LEPT state in triad **1** are shown in Figure 2.

Transient absorption and fluorescence experiments later confirmed this theoretical prediction of an alternative pathway corresponding to a transition from the LES to the LEPT state. This process was recognized as a distinct reaction called PCEnT, where EnT between the anthracene and the phenol−pyridine complex is coupled to proton transfer between the phenol and the pyridine.^21^ The discovery of PCEnT prompted the formulation of a nonadiabatic PCEnT rate theory,^22^ the application of this theory to triad **1**,^23^ and the development of a general form of the PCEnT vibronic coupling that can be decomposed into Förster and Dexter terms.^24^

Despite these advances in our understanding of PCET and PCEnT in the triads,^35^ several key questions still remain. The most important remaining question is, what are the nuclear reaction coordinates that drive PCET and PCEnT, besides the transfer of the proton from phenol to pyridine, and guide the nonadiabatic electronic processes? Understanding how nuclear motions couple to electronic processes is crucial in several contexts. First, it is important for revealing how nature achieves such efficient mastery of charge transfer and energy conversion within existing biological systems. Second, it is important for designing synthetic systems with optimized energetic and electronic flow patterns. As explained below, however, identifying such nuclear reaction coordinates is challenging for nonadiabatic processes, especially for large molecules that are able to follow multiple reaction pathways.

In this work, we address this critical question and identify the key nuclear reaction coordinates in the three triads shown in Figure 1. For this purpose, we use a recently developed computational method^36^ that allows us to explore the range of geometries for which two diabatic electronic states (i.e., LES and CSS or LES and LEPT) have equal energies. Analysis of these geometries enables us to deduce the key effective nuclear reaction coordinates. In particular, we find that the reaction coordinate for PCET corresponds to nuclear motions mainly in the phenol and anthracene but not the pyridine, and the reaction coordinate for PCEnT corresponds to nuclear motions throughout the entire molecule, including the proton acceptor nitrogen in the pyridine.

In addition to identifying the nuclear reaction coordinates, these calculations provide other mechanistic insights. Our analysis suggests different degrees of asynchronicity of ET/EnT and proton transfer, although all of these photochemical processes are concerted without a thermodynamically stable intermediate. Moreover, a quantum mechanical treatment of the transferring hydrogen nucleus to describe hydrogen tunneling suggests different hydrogen tunneling probabilities for PCET and PCEnT, which in turn implicates different kinetic isotope effects. Furthermore, we observe that while both PCET and PCEnT violate the Condon approximation, which assumes that the diabatic electronic coupling is independent of molecular geometry, PCET in the triads passes through a conical intersection in its minimum energy pathway, whereas PCEnT proceeds through an avoided crossing. The generality of these distinctions beyond the triads studied here is not clear, but they do suggest fundamentally different mechanisms for PCET and PCEnT in the triads. The analysis presented herein will guide future studies of PCET and PCEnT, as well as other photochemical processes involving charge and energy transfer.

## 2 Theory and Computational Methods

### 2.1 Defining a Reaction Coordinate via Diabatic States

For thermal reactions occurring on the electronic ground state, a nuclear reaction coordinate with clear physical meaning can often be defined. For example, in an *S*_*N*_2 reaction, the reaction coordinate is the distance between the substrate and the leaving group.^37^ In contrast, because PCET and PCEnT processes in the triads are nonadiabatic and involve multiple excited-state surfaces, finding a physically meaningful nuclear reaction coordinate is more complicated given that most first-principles nonadiabatic dynamics simulations are performed in an adiabatic basis.

The standard resolution to the conundrum above is to invoke a diabatic picture of ET and EnT. Many decades ago, Marcus^38–40^ postulated that the diabatic energy gap can be used as a reaction coordinate for ET. Given the time scale separation between electrons and nuclei, the crossing point between the diabats becomes critical for understanding an ET process. However, it is crucial to emphasize that this crossing point between diabats for a nonlinear molecule is really a seam of dimension 3*N*–7, where *N* is the number of atoms, and practical questions still remain if we seek to map the diabatic energy gap to a nuclear reaction coordinate. For systems such as the triads, there is a possibility that we can isolate an important set of intramolecular nuclear displacements driving the reaction, neglecting the solvent degrees of freedom, which are known to also contribute to the reaction coordinate for PCET.^27^ We hypothesize that such an intramolecular nuclear reaction coordinate can be found by evaluating the nuclear gradient of the diabatic energy gap at the diabatic minimum energy crossing point (DMECP), the minimum energy point in the diabatic seam space. This gradient is defined as

1 $$g_{\mathrm{DMECP}}=\nabla_{R}\left(E_{D}-E_{A}\right){|}_{R=R_{\mathrm{DMECP}}}$$

Here, *E*_D_ and *E*_A_ are the energies of the donor and acceptor diabats, and **R** is the position vector of the 3*N* nuclear coordinates. Equation 1 corresponds to the direction that moves the system from the donor to the acceptor diabat, and we hypothesize that it provides a zeroth-order estimate of the nuclear reaction coordinate.

### 2.2 Constructing Diabatic States

Because electronic structure packages compute adiabatic (rather than diabatic) observables, constructing diabatic states from adiabatic states is of central importance if we seek to use eq 1.^41–48^ Although rigorously exact diabatic states cannot be achieved,^49^ adiabatic states |Φ⟩ can be rotated into *quasi*-diabatic states |Ξ⟩. In this manner, we rotate the Hamiltonian from an adiabatic basis (indexed by 1 and 2) to a diabatic basis (indexed by D and A)

2 $$\left|\Xi_{i}\right\rangle =\sum_{j=1}^{N_{\mathrm{states}}}{\left(U\right)}_{\mathrm{ji}}\left|\Phi_{j}\right\rangle$$

3 $$U^{†}\left(\begin{array}{ll} E_{1}\left(R\right) & 0\\ 0 & E_{2}\left(R\right) \end{array}\right)U=\left(\begin{array}{ll} E_{D}\left(R\right) & H_{\mathrm{DA}}\\ H_{\mathrm{DA}} & E_{A}\left(R\right) \end{array}\right)$$

The off-diagonal term *H*_DA_ is the diabatic electronic coupling, which is an important quantity for calculating the rate constants of nonadiabatic ET in Marcus Theory^38–40^ and vibronically nonadiabatic reactions such as PCET^19,30,31,50^ and PCEnT.^22^ The variation of this diabatic electronic coupling within the seam space is critical because it can indicate adherence (or lack thereof) to the Condon approximation^36^ or an intersection of adiabats, such as a conical intersection, as discussed below.

In order to specify **U**, we can locally disentangle a small number of electronic states by diagonalizing an electronic observable, which leads to an adiabatic-to-diabatic transformation. In general, **U** is clearly not unique, but results with one **U** are often similar to results with another **U** for different physically reasonable schemes. For instance, recent calculations of vibronic couplings for PCEnT in the triads produced similar results using BoysOV diabatization versus Edmiston-Ruedenberg (ER) diabatization.^24^ In this study, we use two slightly different diabatization techniques for PCET and PCEnT. For PCET, where our focus is on ET, we use Boys diabatization to choose the matrix **U** such that the diabatic states have maximally distinct dipole moments (i.e., localized charge). For EnT processes such as PCEnT, Boys diabatization is not adequate because the LES and LEPT states have similar dipole moments. In this case, we employ BoysOV diabatization, which produces diabats with maximally different occupied (hole) and virtual (particle) dipole moments. We discuss the mathematical details of these diabatization techniques and provide a comparison of the diabatic energy gaps and diabatic electronic couplings computed with different diabatization schemes in Sections S1.1 and S2.2 of the Supporting Information.

### 2.3 E-SHAKE for Diabatic Seam Sampling

For model problems with harmonic diabatic potential energy surfaces, the seam of diabatic crossing is a flat hyperplane. For realistic systems studied via first-principles simulations, however, the diabatic seam can be far more complicated and must be generated numerically. To that end, the E-SHAKE method^36,51^ allows us to run Born–Oppenheimer molecular dynamics constrained to a diabatic seam. Mathematically, for a system with *N* nuclei and a 3*N*–6 dimensional internal configurational space, we run dynamics along an adiabatic surface *V*(**R**), where **R** is confined to a 3*N*–7 dimensional seam space.

To achieve such a dynamical routine, we propagate Newton’s equations of motion along an adiabatic surface *V* but with a constraint force

4 $$\mathrm{R̈}=-M^{-1}\nabla_{R}V+M^{-1}F_{C}$$

Here, **M** is a matrix of size 3*N* with the mass of each atom on the diagonal, the first term on the right-hand side is the usual Newton force, and **F**_C_ is the constraint force. To derive the form of **F**_C_, consider the constraint that the diabatic donor and acceptor energy surfaces during molecular dynamics are degenerate

5 $$\sigma \left(t\right)=\sigma \left(R\left(t\right)\right)=E_{D}\left(R\left(t\right)\right)-E_{A}\left(R\left(t\right)\right)=0$$

A form for **F**_C_ can be found by insisting that we not only stay in the seam, but we also never leave the seam, so that

6 $$\mathrm{\sigma ̇}\left(t\right)=Ṙ\cdot \nabla_{R}\sigma =0$$

Satisfying eqs 5 and 6 is a well-known problem in molecular dynamics, with well-known solutions, e.g., the SHAKE and RATTLE algorithms.^52^ In this work, we used a donor–acceptor energy gap threshold of |σ­(**R**)| ≤ ϵ = 4 × 10^–5^ a.u. ≈ 1 meV. In principle, the dynamics described by eq 4 (with damping) could be used to find the minimum energy point in the diabatic seam space, i.e., the DMECP. For larger systems such as those studied herein, we define the DMECP as the point on the seam with the lowest energy sampled during low-temperature simulations. The advantage of analyzing the DMECP via E-SHAKE as opposed to a direct minimization^53^ is that we obtain valuable information about the system in the seam during the sampling process, including the variation of its diabatic electronic coupling and its proximity to a conical intersection.

We used the E-SHAKE algorithm as implemented in INAQS^54^/GROMACS 4.5.^55–58^ All trajectories were started in the vicinity of an avoided crossing and propagated using Born–Oppenheimer molecular dynamics on the lower of the two nearly crossing adiabatic surfaces. The time step for these trajectories was 20 a.u. ≈ 0.5 fs, and the classical dynamics of the nuclei were carried out with the velocity Verlet algorithm.^59^ We used thermostat temperatures of 1, 5, 10, 20, 35, and 50 K, which are similar to those used in previous E-SHAKE simulations.^36^ These temperatures are not physically meaningful but rather are chosen to ensure sufficient sampling of the diabatic seam space. The trajectories were thermostated using the stochastic Berendsen thermostat^60^ with a time constant of τ = 10 fs, with the exception that one trajectory for triad **4** used a time constant of τ = 1 fs for tighter temperature control to find the DMECP. After the DMECP geometries were found, they were plotted with their corresponding reaction coordinates using the VMD software package.^61^ The trajectories presented herein sampled energies up to at least 500 meV above the DMECP and thus should capture the energetically accessible regions of the seam traversed in room temperature nonadiabatic dynamics simulations.

### 2.4 Proton Potentials and Vibrational Wave Functions

To understand the quantum nature of the transferring proton for PCET and PCEnT, we constructed proton potentials and vibrational wave functions. Starting with a DMECP geometry obtained from E-SHAKE, we generated a one-dimensional proton transfer coordinate axis by optimizing the proton on the donor or acceptor on the lower adiabatic surface with all other atoms fixed and then connecting the two optimized positions with a line. We subsequently moved the proton along a grid of 20 evenly spaced points along this proton coordinate axis and performed single-point TDDFT calculations to generate energies for the proton potential energy profiles. The LES, CSS, and LEPT state were identified by examining the electron and hole populations on each subunit of the triad for the adiabatic states. The energies of these diabatic states were assumed to be the same as the adiabatic energies. This procedure is valid far from the crossing point because the diabatic and adiabatic states are essentially equivalent but is not valid near the crossing point as diabatic states of different character mix in this region. In this case, BoysOV diabatization was applied when the states of interest were close to each other, and the calculated diabatic energies were used instead. The proton potential energy profiles were generated from these energies using a B-spline interpolation. Finally, we computed hydrogen and deuterium vibrational wave functions for each proton potential using the Fourier Grid Hamiltonian method.^62^ The overlap integrals between vibrational wave functions for different diabatic states were computed numerically.

### 2.5 Conical Intersections

Although the focus of the discussion above has been on the DMECP, it should be noted that, as far as nonadiabatic reactions are concerned, very few published papers have focused on the DMECP, mainly because diabatic states are not unique and diabatic gradients^63–66^ are not available in most quantum chemistry codes. Most computational studies of ET and EnT focus on the minimum energy crossing point (MECP),^53^ which can be found by minimizing the energy of the conical intersection seam between adiabats.^67^ Once thought to be rare, conical intersections are now known to be ubiquitous in photochemistry^68^ and to significantly impact the rates of photochemical reactions.^69–72^ The 3*N*–8 dimensional conical intersection seam is contained within the diabatic seam, with the additional constraint that *H*_DA_(**R**) = 0.

In a diabatic basis, the two coordinates that lift the degeneracy of the conical intersection, or the vectors that define the “branching plane,” are the **g** and **h** vectors^68^ defined as

7 $$g=\nabla_{R}\left(E_{D}-E_{A}\right)=\nabla_{R}\sigma$$

8 $$h=\nabla_{R}H_{\mathrm{DA}}$$

To confirm that an intersection of adiabatic states is indeed conical, the relevant geometric phase can be calculated. Given the derivative coupling between adiabatic states 1 and 2,

9 $$d_{12}=\left\langle \Phi_{1}\right|\nabla_{R}\left|\Phi_{2}\right\rangle$$

a simple two-state diabatic model predicts that the line integral of this vector around a conical intersection in its branching plane evaluates to ±π^73^

10 $$\oint d_{12}\cdot dl=\pm \pi$$

Here, the path in the line integral is defined as

11 $$l=r_{\mathrm{int}}+r_{g}\cos\left(\theta \right)g+r_{h}\sin\left(\theta \right)h$$

For our calculations, we began our analysis by isolating two points in the diabatic seam space with diabatic couplings of opposite signs and then approximating **r**_int_ by interpolation. Next, we computed the derivative couplings along the path in eq 11, making sure we aligned the phases of the derivative couplings from point to point. We confirmed that the gap between adiabatic energies *E*_2_ and *E*_1_ never vanished along the path, and then we evaluated the integral in eq 10. All calculations were performed at the TDDFT level. Because our calculations never involved the ground state, we did not need to invoke more sophisticated approaches.^74–76^

### 2.6 DFT Electronic Structure Details

The electronic structure calculations for the triads were performed using the Tamm−Dancoff approximation to time-dependent density functional theory (TDA-TDDFT) in the Q-CHEM 6.2 package.^77^ The proton potential calculations for triad **1** were conducted with the same level of theory but with Q-CHEM 6.4 and the pyPCET package.^78^ The CAM-B3LYP functional^79^ was used with the 6-31G­(d,p)^80–82^ basis set for all calculations. This functional was chosen because of its agreement with higher-level complete active space self-consistent field with N-electron valence state perturbation theory (CASSCF-NEVPT2) calculations of the triads in previous studies.^33,34^ Additional calculations for PCET in triad **1** and PCEnT in triad **4** were performed with the ωB97X-D functional^83^ and are provided in the Supporting Information. No major qualitative differences were observed between the results obtained with these two functionals.

## 3 Results and Discussion

### 3.1 Diabatic Electronic Coupling for PCET and PCEnT

We begin our analysis by analyzing the diabatic electronic couplings obtained during the seam sampling for the PCET and PCEnT reactions. For all reactions studied, the reactant state is the LES. The *S*_1_ surface is of LES character for the geometries sampled in the diabatic seam for both PCET reactions and for PCEnT in triad **4**, but the *S*_1_ surface is of CSS character and the *S*_2_ surface is of LES character for the geometries sampled in the diabatic seam for PCEnT in triad **1**. Thus, PCET occurs when the *S*_1_ adiabatic surface of triad **1** or **3** changes character from LES to CSS, and PCEnT occurs when the *S*_2_ surface of triad **1** or the *S*_1_ surface of triad **4** changes character from LES to LEPT. Here *S*_1_ and *S*_2_ refer to the first and second excited electronic states, respectively. The diabatic electronic couplings were computed during the constrained molecular dynamics in the seam space, where the energies of the two diabatic states are the same to within a specified threshold. Figure 3 shows the diabatic electronic coupling *H*_DA_ between the LES and CSS for PCET (or the LES and LEPT state for PCEnT) versus the energy of the lower adiabatic surface for all four systems.

**Figure 3:**
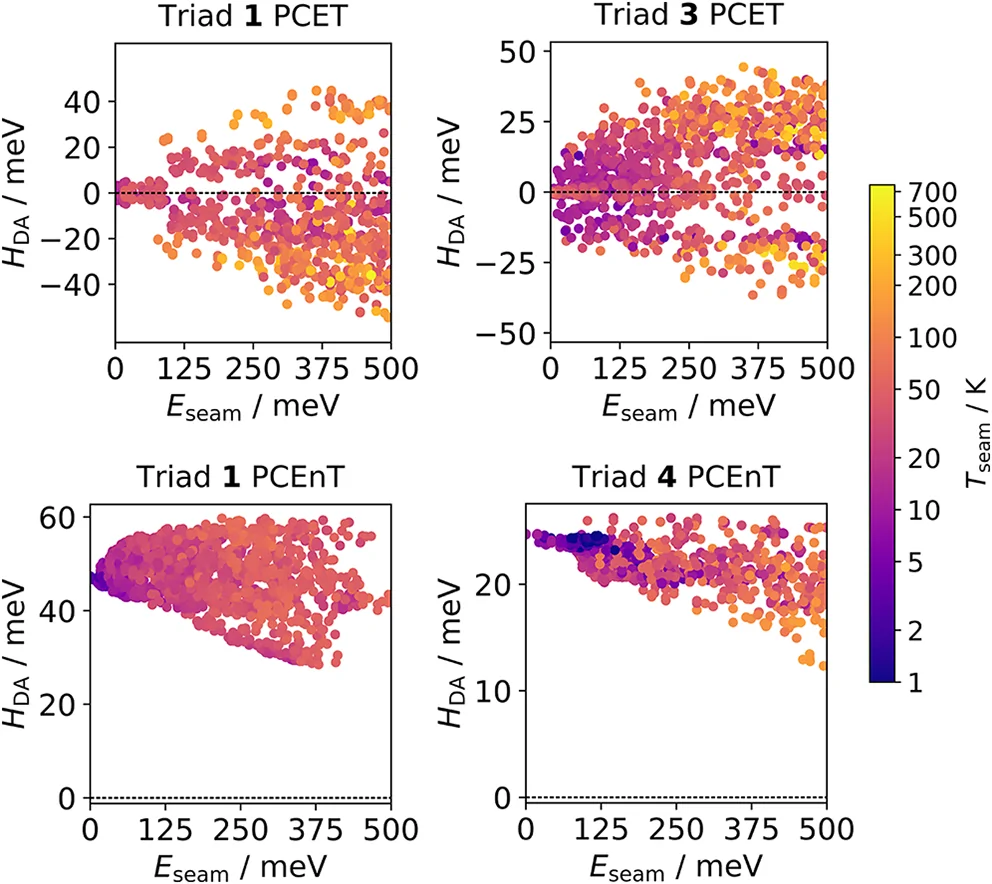
Diabatic electronic couplings between the LES and CSS for PCET or between the LES and LEPT state for PCEnT versus the energy of its lower adiabatic surface relative to its DMECP. The color of each point is determined by the instantaneous temperature during the diabatic seam sampling. All diabatic seams appear to violate the Condon approximation in that the diabatic electronic coupling depends on the nuclear coordinates (i.e., it changes in the seam space). Only the LES–CSS diabatic coupling associated with PCET passes through zero in our seam sampling, indicating an energetically relevant intersection of adiabatic surfaces in the diabatic seam.

The sign of the diabatic coupling was phase-matched for all systems by ensuring that the off-diagonal matrix element of the dipole operator in the diabatic representation was continuous. The color of each point indicates the instantaneous temperature, as determined by the kinetic energy. The black dotted line corresponds to zero diabatic coupling, which indicates an intersection of adiabatic surfaces.

All four reactions examined with seam sampling exhibit large ranges of diabatic electronic coupling. In particular, for geometries sampled up to 500 meV relative to the DMECP energy, the standard deviations were 19 and 17 meV for the PCET reactions and 7 and 2 meV for the PCEnT reactions. These types of non-Condon effects, where the diabatic coupling depends strongly on the nuclear coordinates, are typical for large floppy molecules such as the triads. Notably, we observe a marked difference between the spreads of the diabatic electronic couplings for the PCET versus the PCEnT reactions. We discuss these non-Condon effects further in the Supporting Information.

One surprising difference between the seam sampling results for PCET and PCEnT is whether or not the diabatic coupling passes through zero. The diabatic couplings change sign even after phase-matching for both PCET reactions, whereas they do not change sign in either PCEnT reaction. Furthermore, these sign changes for the PCET systems occur near the DMECP (i.e., when *E*_seam_ ≈ 0), revealing a chemically relevant *adiabatic* surface intersection within the diabatic seam space that exists for PCET but not for PCEnT. Our seam sampling analysis herein does not preclude the existence of adiabatic intersections for PCEnT, but rather suggests that these intersections are high in energy relative to the DMECP and thus are less likely to be sampled during the PCEnT reaction. In the next subsection, we present the analysis described in Section 2.5 to determine whether or not the intersections of adiabatic states in the LES–CSS seams are conical.

### 3.2 Determining the Existence of a Conical Intersection

As discussed above, the diabatic electronic coupling passes through zero close to the DMECP for PCET but not for PCEnT, suggesting the possibility of a low-lying conical intersection for PCET. Equipped with the DMECP geometries for the PCET reactions, we now rigorously demonstrate the existence of a physically meaningful conical intersection for these reactions. We found our putative conical intersection geometry by linearly interpolating between the DMECP geometry and another geometry in the diabatic seam to find a geometry with nearly zero diabatic coupling. We subsequently computed the derivative coupling between the *S*_1_ and *S*_2_ states at 50 points within the branching plane and evaluated the line integral numerically at every step (Figures 4 and S5). For both PCET reactions, the magnitude of **g** was significantly larger than that of **h**, so we chose *r*_*g*_ = 2.5 a.u. and *r*_*h*_ = 75 a.u. to ensure a sufficient energy gap between the *S*_1_ and *S*_2_ states. We find clear evidence of a conical intersection for PCET in triads **1** and **3**, with geometric phases of ≈ −1.04π and ≈ −1.03 π, respectively.

**Figure 4:**
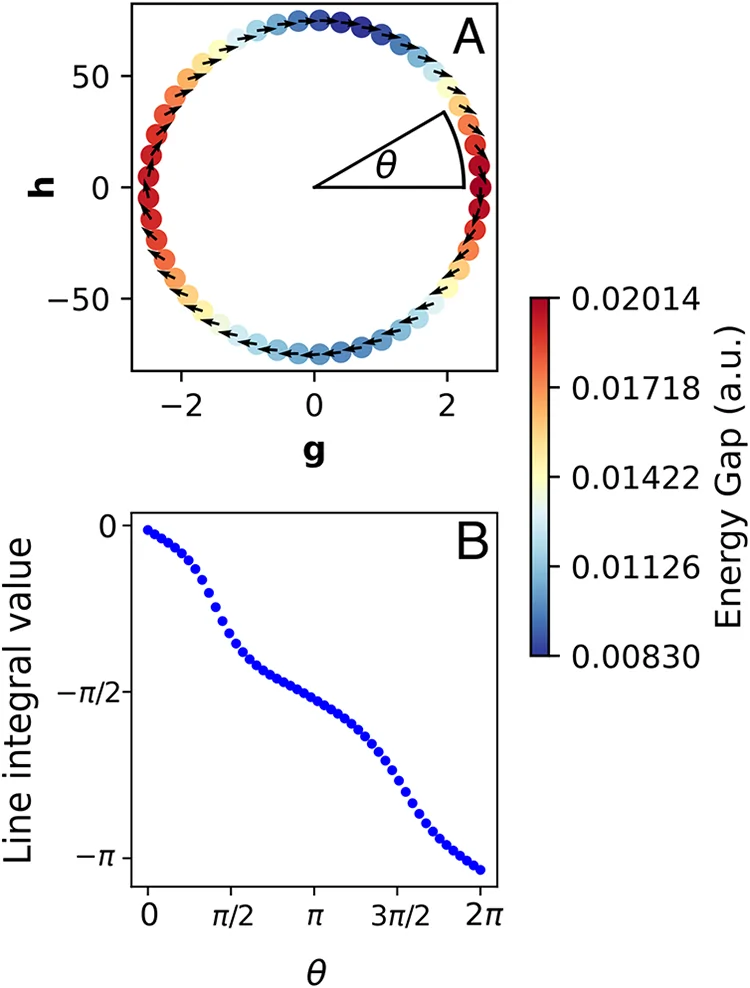
(A) Depiction of the triad **1** branching plane with the **g** and **h** vectors as axes. Each point is a single-point TDDFT calculation along the path given by eq 11 and is colored by the magnitude of the energy gap between the *S*_1_ and *S*_2_ adiabatic surfaces. The arrow on each point is the projection of the derivative coupling vector in the branching plane. A nonzero energy gap opens up in both the **g** and **h** directions, and the derivative coupling varies smoothly, indicating a suitable path for the line integral. The angle of integration θ for the line integral is also shown. (B) The value of the line integral evaluated from θ = 0 along the positive *x*-axis to the angle θ measured relative to the *x*-axis. The integral evaluates to –1.04π for triad **1**, proving that the intersection of adiabats is indeed conical. Almost identical results were found for triad **3**, as shown in Figure S5.

Note that the conical intersection points around which Figures 4 and S5 were evaluated are not rigorous minimum energy points within the adiabatic intersection spaces of these systems but rather provide upper bounds to the energy gap between the MECP and DMECP. Indeed, the MECP and DMECP cannot coincide for clear geometric reasons; the DMECP has a branching plane of codimension 1 and the MECP has a branching plane of codimension 2. Nevertheless, we find the energy gap between the DMECP and the interpolated conical intersection geometry to be 1.12 kcal/mol for triad **1** and 0.03 kcal/mol for triad **3**. These small values suggest that the presence of a conical intersection will have significant effects on the dynamics of the PCET reactions. In other words, photochemical PCET in triads **1** and **3** violates the tenets of Marcus theory not only by allowing for fluctuations in the diabatic coupling (i.e., disobeying the Condon approximation) but also by exhibiting an average diabatic electronic coupling close to zero for the geometries of interest. More advanced calculations^84,85^ will be needed in the future to compute rigorous PCET rate constants and kinetic isotope effects in these systems.

### 3.3 DMECP Geometries and Reaction Coordinates for PCET and PCEnT

We next examine the DMECP geometry and the reaction coordinate defined in eq 1 of each system as found by seam sampling. Figure 5 shows the DMECP geometries and the reaction coordinates for PCET and PCEnT indicated by atom-resolved vectors with lengths proportional to the magnitudes of the contributions. Here, we analyze the similarities and differences between the reaction coordinates for the two processes, corresponding to charge transfer versus energy transfer.

**Figure 5:**
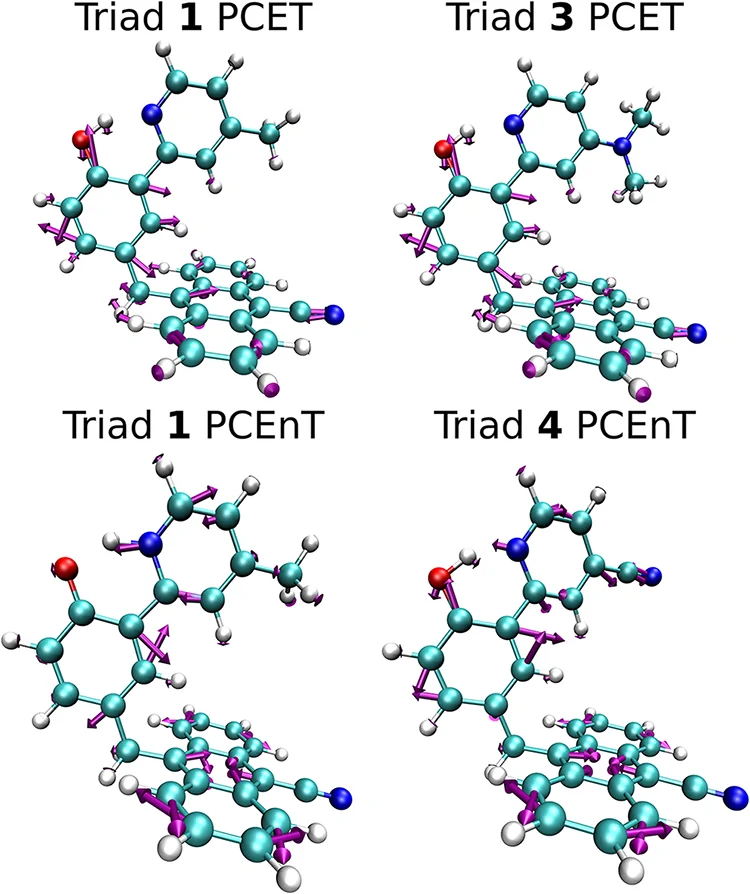
DMECP geometries for triads **1** and **3** in the LES–CSS seam and triads **1** and **4** in the LES–LEPT seam with the reaction coordinate vectors shown on each atom in purple. The relative lengths of the vectors indicate the relative magnitudes of the contributions of those motions to the reaction coordinate. These motions are driving the PCET and PCEnT reactions, corresponding to changing charge densities and proton transfer from the phenol to the pyridine. The motion of the proton is more aligned with the donor–acceptor axis in PCEnT than in PCET. The PCEnT reaction coordinates have significant contributions from the pyridine subunit, including the proton acceptor, whereas the PCET reaction coordinates lack these contributions.

The PCET reaction coordinates contain significant atom-resolved vectors on the phenol and anthracene fragments, but not the pyridine fragment. In contrast, significant atom-resolved vectors appear on all three fragments in the PCEnT reaction coordinates. We can quantify the relative contributions from each fragment of the triad to the reaction coordinate by computing the sum of squared components of the normalized atom-resolved vectors. In the case of triad **1**, these relative contributions (on a scale from 0 to 1) on the phenol, anthracene, and pyridine fragments of the molecule, respectively, are 0.443, 0.486, and 0.031 for PCET and 0.149, 0.690, and 0.150 for PCEnT. A similar pattern holds for triads **3** and **4** as well. These numbers do not sum to 1 because the transferring proton and the methylene spacer between phenol and anthracene were not included. This finding indicates that the nuclear motions of the pyridine are less coupled to PCET than to PCEnT. This result is consistent with previous assertions^21,23^ that pyridine is not a significant electron donor or acceptor in PCET but is a significant energy acceptor in PCEnT alongside phenol.

The directions of the atom-resolved vectors, while slightly different for each system, are qualitatively similar for the systems undergoing the same reaction. These common features thus appear to be characteristics of the reaction coordinates for either PCET or PCEnT in the triads. Both the PCET and PCEnT reaction coordinates show a contraction of the C–O bond associated with proton transfer from the oxygen to the nitrogen. In particular, the reaction coordinate for PCET in triads **1** and **3** and the reaction coordinate for PCEnT in triad **4** have large vectors on the C and O pointing toward each other. (PCEnT in triad **1** already has a C–O double bond at its DMECP because the proton has already transferred.) However, some differences between the PCET and PCEnT reaction coordinates are apparent in the C–C bond stretches and contractions throughout the aromatic rings of phenol and anthracene, as well as the cyano group.

The differences between the PCET and PCEnT reaction coordinates are mainly due to the distinct changes in charge density upon ET or EnT. The charge on the anthracene fragment is virtually zero for the LES and LEPT state but is −1 for the CSS, corresponding to ET from phenol to anthracene. Moreover, the total charge on the phenol−pyridine complex is virtually zero for the LES and LEPT state but is +1 for the CSS. Upon further decomposition, the charges on the phenol and pyridine fragments are both virtually zero for the LES but are slightly charged for the LEPT state, corresponding to a small amount of electronic charge rearrangement.^21^ Consistent with these differences in charge distributions, the directions of the vectors on the carbon atoms in the aromatic rings of the anthracene are quite different between PCET and PCEnT. The PCET reactions only exhibit one significant vector on the anthracene’s central aromatic ring and vectors that point directly opposite each other on the anthracene’s peripheral carbon atoms, as well as vectors on the cyano group C–N atoms pointing toward each other. In contrast, the PCEnT reactions exhibit six significant vectors on the central aromatic ring pointing inward and vectors that point in different directions on the peripheral carbon atoms, without any significant motion within the cyano group. These qualitative differences between the reaction coordinate vectors on the anthracene fragment are consistent with a significantly different final state for anthracene after ET than after EnT: the PCET product corresponds to an anthracene anion, whereas the PCEnT product corresponds to a neutral anthracene in its ground state.

### 3.4 Synchronicity and Asynchronicity of ET/EnT with Proton Transfer

Both PCET and PCEnT in the triads have been shown experimentally and theoretically to be concerted, in that they occur without a thermodynamically stable intermediate associated with either ET/EnT or proton transfer.^21,27^ In principle, however, ET/EnT and proton transfer could be asynchronous in that they do not occur at exactly the same time, separated by femtoseconds or tens of femtoseconds.^86^ Previous nonequilibrium excited state dynamics simulations with a classical proton suggested that proton transfer occurs very quickly after ET for triads **1** and **3** and after EnT for triad **4**, although the time scales for these trajectories were not meaningful because they were propagated at high temperatures.^34^

We emphasize that analyses of asynchronicity based on a classical treatment of the transferring proton should be viewed with caution given that previous work has shown the importance of treating the transferring proton quantum mechanically and incorporating hydrogen tunneling effects. However, such analyses can still provide useful insights from an alternative perspective. This analysis will be placed in the context of vibronically nonadiabatic theories that include hydrogen tunneling^19,30^ in the next subsection.

Our reaction coordinate analysis may shed light on the degree of asynchronicity for PCET and PCEnT. The contributions of the proton donor and acceptor motion to the reaction coordinate can elucidate whether these motions are coupled to ET or EnT. Additionally, the position of the proton at the DMECP can provide valuable insight as to whether proton transfer occurs before or after the system has reached the seam space. However, these findings should be considered with the caveat that we are not running direct dynamics, which can be strongly influenced by initial conditions.^87^

To begin our analysis, consider the role of the transferring proton, as well as the proton donor and acceptor, in the PCET and PCEnT reaction coordinates. We can quantify the motion of the proton from the donor atom to the acceptor atom by calculating the normalized projection of the reaction coordinate vector associated with the transferring proton onto the donor–acceptor axis (a straight line connecting the donor to the acceptor). In both PCET reactions, the reaction coordinates exhibit a “wagging” of the enol O–H bond that is not toward the acceptor nitrogen atom but rather corresponds to a bending motion, with projections of the proton motion onto the donor–acceptor axis of −0.22 for both triads **1** and **3**. The acceptor nitrogen motion does not contribute significantly to either PCET reaction coordinate. In contrast, both PCEnT reaction coordinates show proton motion aligned with the donor–acceptor axis, with projections of the proton motion onto the donor–acceptor axis of 0.99 for triad **1** and 0.51 for triad **4**. Furthermore, the motion of the acceptor nitrogen has a much stronger contribution to these reaction coordinates.

Next, we consider the position of the transferring proton at the DMECP and throughout the seam space. At the DMECP geometries for the PCET reactions, the proton is closer to the donor oxygen atom than it is to the acceptor nitrogen atom, with O–H/N–H distances of 1.02 Å/1.56 Å and 1.02 Å/1.59 Å for triads **1** and **3**, respectively, indicating that the proton remains bound to the donor oxygen atom. However, the transferring protons have different positions in the two DMECP geometries for the PCEnT reactions, with O–H/N–H distances of 1.49 Å/1.10 Å and 1.06 Å/1.51 Å for triads **1** and **4**, respectively, indicating that the proton remains bound to the donor oxygen atom for triad **4** but has transferred to the acceptor nitrogen atom for triad **1**.

To further analyze the position of the proton during PCET or PCEnT, in Figure 6 we plot the difference between the distance of the proton to its donor and to its acceptor (*R*_OH_ – *R*_NH_) versus the energy of the adiabatic surface used for the seam sampling. The black dashed line indicates where the proton is equidistant from the donor and acceptor atoms. For PCET in triads **1** and **3** and PCEnT in triad **4**, despite some spread in *R*_OH_ – *R*_NH_, the proton remains bound to the donor oxygen, as observed for the DMECP geometries. Although there may be geometries in these diabatic seams where the proton is bound to the acceptor nitrogen, such geometries were not found during seam sampling and are most likely very high in energy. This result suggests that ET or EnT is likely to occur prior to proton transfer for these systems, although the asynchronicity may only be on the order of femtoseconds. This picture is different for PCEnT in triad **1**: *R*_OH_ – *R*_NH_ > 0 for most geometries (including the DMECP geometry), indicating that the proton has transferred to the nitrogen, but *R*_OH_ – *R*_NH_ < 0 for some geometries, starting at 250 meV above the DMECP, indicating that EnT can still occur with the proton bound to the phenolic oxygen at energies reasonably close to the DMECP. In this case, the proton transfer could occur either before or after EnT, where again the asynchronicity could be on the order of femtoseconds.

**Figure 6:**
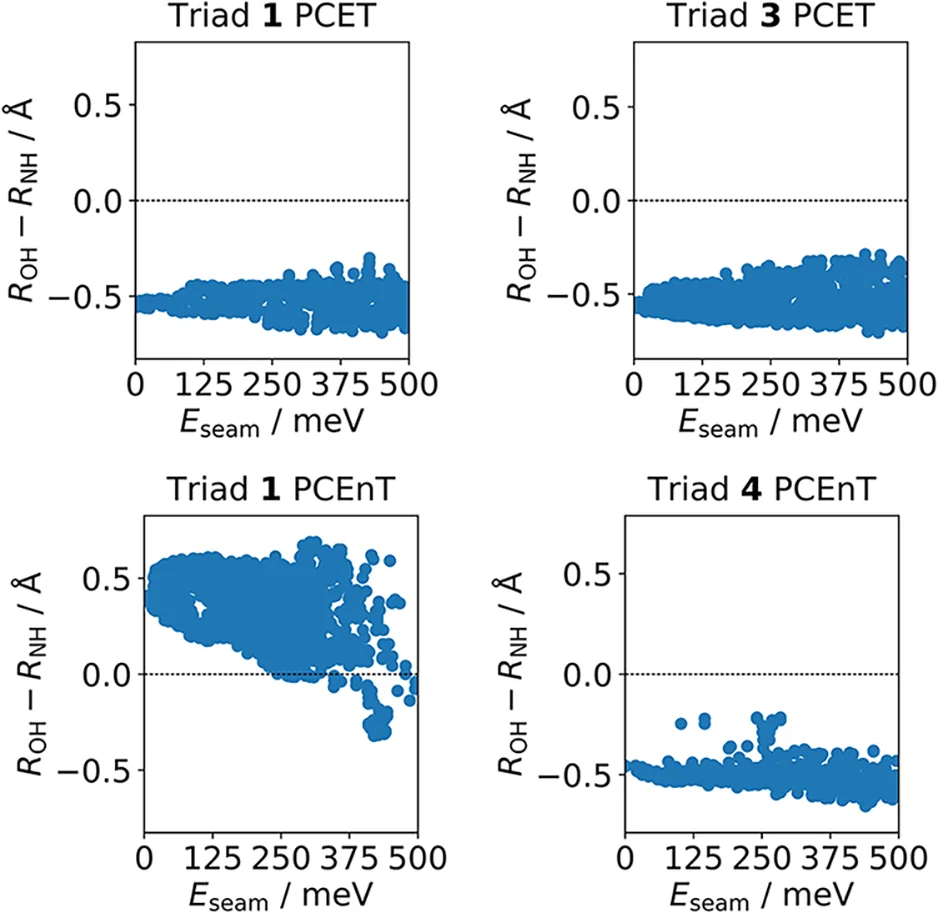
Plots of *R*_OH_ – *R*_NH_ versus the seam energy *E*_seam_ for each system studied. *R*_OH_ −*R*_NH_ < 0 indicates that the proton is closer to the donor oxygen, and *R*_OH_ – *R*_NH_ > 0 indicates that the proton is closer to the acceptor nitrogen. Although this proton coordinate varies within each diabatic seam, the LES–CSS seams and the LES–LEPT seam in triad **4** exhibit only negative *R*_OH_ – *R*_NH_ values, whereas the LES–LEPT seam in triad **1** exhibits both positive and negative *R*_OH_ – *R*_NH_ values.

This combined analysis suggests that both PCET and PCEnT in the triads may be asynchronous, but PCEnT may be more synchronous than PCET. Two characteristics of the PCEnT reaction coordinates supporting this distinction are that the proton motion is more aligned with the donor–acceptor axis and that the proton acceptor nitrogen motion toward the transferring hydrogen contributes more significantly. Additionally, for one of the PCEnT reactions studied, the proton can transfer back and forth during the seam sampling. However, this analysis does not provide information about the time scale associated with the asynchronicity of either PCET or PCEnT (i.e., the time between ET/EnT and proton transfer). A full analysis of asynchronicity in the context of a classical treatment of the transferring proton would require the propagation of nonadiabatic dynamics trajectories sampled over an ensemble of initial conditions. The analysis of asynchronicity in the context of a quantum treatment of the transferring proton is discussed in the framework of vibronically nonadiabatic theories in the next subsection.

### 3.5 Hydrogen Tunneling and Kinetic Isotope Effects

Our calculations thus far have neglected the quantum mechanical nature of the transferring proton. Hydrogen tunneling has been shown to play an important role in both PCET and PCEnT for these triads.^23,27^ The vibronically nonadiabatic theories of PCET^19,30^ and PCEnT^22^ describe these reactions in terms of nonadiabatic transitions between pairs of reactant and product diabatic vibronic states. Each diabatic vibronic state is the product of a diabatic electronic state and a corresponding proton vibrational state. The nonadiabatic rate constants for both PCET and PCEnT are expressed as a sum of rate constants associated with each pair of vibronic states, where each term is proportional to the square of the vibronic coupling. For many systems, such as the triads, the vibronic coupling is approximately the product of the diabatic electronic coupling and the overlap integral between the reactant and product proton vibrational wave functions.^24,30,88,89^ Each term also depends on other factors, which differ for PCET versus PCEnT.

The mechanism underlying these vibronically nonadiabatic theories is that fluctuations of the solute and/or solvent lead to the degeneracy of a pair of reactant and product vibronic states. At this configuration, the probability of a transition between vibronic states is proportional to the square of the vibronic coupling. To understand how the concepts of asynchronicity and synchronicity can arise in this framework, consider a general PCET reaction, where the electron is localized on its donor in all reactant vibronic states and on its acceptor in all product vibronic states. If the proton is localized near its donor in the reactant vibronic state and near its acceptor in the product vibronic state, both the electron and proton tunnel simultaneously from their donors to their acceptors during the nonadiabatic transition. In this case, the PCET reaction must be synchronous. However, for some excited product vibronic states, the proton may be localized near its donor and will eventually relax to its ground proton vibrational state localized near its acceptor. In this case, the PCET reaction can be viewed as asynchronous in the sense that the electron will tunnel prior to proton transfer. The analysis of asynchronicity within vibronically nonadiabatic theories can be accomplished by computing the proton position expectation value for the dominant nonadiabatic transition(s) or along trajectories propagated with quantum master equations in a basis of vibronic states. Such an analysis, as well as the calculation of PCET and PCEnT rate constants, is beyond the scope of the present work.

Instead, we focus on analyzing the proton potentials and vibrational wave functions at the DMECP geometries produced by our diabatic seam sampling. This analysis provides insight into key differences between PCET and PCEnT. Using our DMECP geometries for PCET and PCEnT in triad **1**, we constructed the proton potential energy profiles and computed the proton vibrational wave functions for the LES, CSS, and LEPT state. Triad **1** was chosen for these calculations because the LES, CSS, and LEPT state are well-separated in energy from other excited states. We calculated these proton potentials for the LES–CSS DMECP geometry associated with PCET and the LES–LEPT DMECP geometry associated with PCEnT. The proton potentials and the vibrational wave functions associated with the two lowest-energy vibrational states for each diabatic electronic state are shown in Figure 7. The proton potential energy profiles are asymmetric: the proton donor well is lower in energy for the LES, and the proton acceptor well is lower in energy for the CSS and LEPT state. These asymmetries show that proton transfer is energetically favorable for the CSS and LEPT state. The CSS proton potential is only slightly asymmetric, and we expect the potential to become even more asymmetric in the presence of a polar solvent that can stabilize the separated charges. The differences in the shapes of the LES proton potentials for PCET and PCEnT are due to differences in the DMECP geometries. In particular, the phenol-pyridine dihedral angle (defined in Figure S1 of the Supporting Information) is 14.1° for PCET and 0.3° for PCEnT.

**Figure 7:**
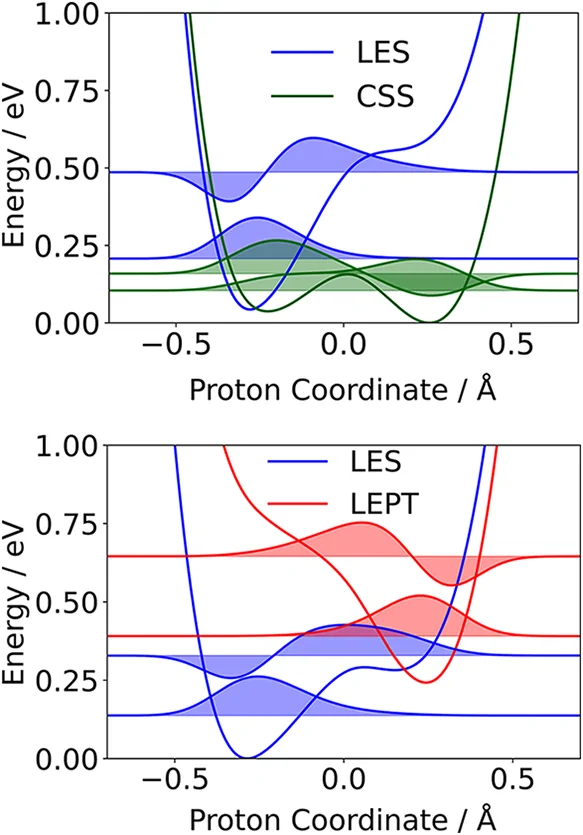
Proton potential energy profiles and H vibrational wave functions for PCET (top) and PCEnT (bottom) in triad **1**. The energy in each plot is relative to the minimum energy of the two proton potentials shown. We show the first two proton vibrational wave functions for each diabatic state. Note that PCET and PCEnT may involve nonadiabatic transitions between vibronic states not shown here.

Next, we calculated the overlap integrals between the reactant and product hydrogen and deuterium vibrational wave functions for PCET and PCEnT. The square of this overlap integral is related to the probability of hydrogen or deuterium tunneling. If only a single pair of vibronic states contributed to the rate constant at a single geometry, the ratio of the squares of the hydrogen and deuterium overlap integrals would provide an estimate of the kinetic isotope effect. Because multiple pairs of vibronic states often contribute to the PCET or PCEnT rate constant, and the dominant pair may differ for hydrogen and deuterium, a single ratio of overlap integrals is not usually sufficient to estimate the kinetic isotope effect.^35^ Moreover, the total rate constant for PCET and PCEnT requires thermal averaging over the proton donor–acceptor distance, rather than a calculation for a single geometry such as the DMECP. Nevertheless, comparison of these ratios of overlap integrals has potential implications for the relative kinetic isotope effects.

For simplicity, we first consider the ratio of overlaps between the lowest energy proton vibrational state for each diabatic electronic state, |*S*_00_^H^|^2^/|*S*_00_^D^|^2^. The PCEnT squared overlap ratio of 21 agrees well with the previously calculated kinetic isotope effect for PCEnT in the same triad, as the (0,0) pair dominates this PCEnT process.^23^ However, the PCET reaction, which has a squared overlap ratio of 5.6, is expected to involve excited vibronic states, which typically lower the kinetic isotope effect, and the dominant pair may be different for H and D. Interestingly, the first excited proton vibrational state for the CSS proton potential energy profile is localized in the donor well. Based on the discussion above, a nonadiabatic transition to this vibronic state could be associated with an asynchronous PCET reaction. All overlap integrals pertaining to the lowest two reactant and product vibronic states are provided in Table S3 of the Supporting Information. Based on the overlap integrals, the (0,1) pair of vibronic states is expected to contribute significantly to PCET for both H and D. This contribution is expected to decrease the kinetic isotope effect, although analysis of the quantitative impact requires the calculation of the full rate constant for H and D. Note that this analysis does not account for the non-Condon effects in the triads (i.e., the dependence of the overlap integral on the nuclear coordinates), although thermal averaging over the proton donor–acceptor distance would address this issue to some extent. Finally, our calculations do not consider the presence of a conical intersection within the LES–CSS seam space, which may preclude the use of the vibronically nonadiabatic PCET rate constant expression based on the golden rule formalism. Nevertheless, this simplified analysis predicts distinct kinetic isotope effects for PCET and PCEnT.

## 4 Conclusions and Future Directions

From these results, we can deduce and predict several features about the reaction coordinates for PCET and PCEnT in the triads. First, the reaction coordinates found for PCET and PCEnT agree with the qualitative understanding of these reactions established previously. The LES–CSS transition in PCET, which involves ET from phenol to anthracene, is coupled most strongly to the nuclear motions in the phenol and anthracene but not the pyridine. In contrast, the LES–LEPT transition in PCEnT, which involves EnT from anthracene to the phenol–pyridine complex, is coupled to nuclear motions throughout the entire triad. Both reaction coordinates have significant contributions associated with contraction of the phenol C–O bond, as well as contributions from carbon atoms on the aromatic rings of anthracene, which is consistent with a change in charge density. However, the latter contributions are different for PCET and PCEnT because the LES of anthracene changes to the anionic state for PCET and to the neutral ground state for PCEnT.

One key difference between the reaction coordinates for PCET and PCEnT is that the proton acceptor motion toward the transferring proton contributes significantly for PCEnT but not for PCET. Moreover, the proton remains bound to the phenol in the diabatic seam for both PCET reactions and for PCEnT in triad **4**, but the proton can transfer between the phenol and the pyridine in the diabatic seam for PCEnT in triad **1**. Although both PCET and PCEnT are concerted, without a stable intermediate, these findings suggest that they may be asynchronous, with ET or EnT most likely occurring prior to proton transfer. These findings also suggest that PCEnT may be more synchronous than PCET. However, this analysis does not provide information about the time scales associated with asynchronicity. This type of information would require propagation of real-time nonadiabatic dynamics trajectories sampling an ensemble of initial conditions. Moreover, this analysis does not account for hydrogen tunneling, which is known to be important in these reactions. A quantum mechanical treatment of the transferring proton to incorporate hydrogen tunneling effects within vibronically nonadiabatic theories of PCET and PCEnT also suggests that PCEnT may be more synchronous than PCET in the gas phase and suggests that the kinetic isotope effects may be greater for PCEnT. A full analysis of the rate constants would be required to confirm this qualitative prediction about the relative kinetic isotope effects. However, this prediction is in line with experimental measurements of moderate kinetic isotope effects for the PCET reactions^27^ as well as a more complete theoretical kinetic analysis of PCEnT for triad **1** predicting a much greater kinetic isotope effect.^23^

In this paper, we have further established that both PCET and PCEnT violate the Condon approximation, as evidenced by large variations in diabatic electronic coupling with respect to changes in nuclear coordinates for all four diabatic seams considered. The PCET reactions exhibit significantly greater fluctuations in these couplings than do the PCEnT reactions. Furthermore, the magnitude of the fluctuations relative to the magnitude of the coupling at the DMECP is small for PCEnT but enormous for PCET because of the presence of a conical intersection for the latter case. This result suggests that vibronically nonadiabatic PCET rate constant calculations effectively invoking the Condon approximation (i.e., allowing the diabatic coupling to depend only on the donor–acceptor distance) may not be applicable to PCET associated with charge separation in the triads.

Our analysis so far has been restricted to the gas phase, but examination of the impact of solvent on the diabatic seams of these molecules warrants further study. In particular, polar solvents will stabilize the CSS more than the LES or LEPT state.^25^ Solvent effects will also lead to more asymmetric proton potential energy profiles for the CSS, influencing the analysis of PCET in the context of vibronically nonadiabatic PCET theory. Additionally, it will be interesting to determine if solvent significantly alters the conical intersection we have predicted for PCET. However, we do not expect solvent to significantly impact the dominant solute motions driving PCET and PCEnT. Overall, this work provides fundamental insights about the differences between PCET and PCEnT photochemical processes. These insights should help guide the design of systems that favor either charge transfer or energy transfer coupled to proton motion.

## Supplementary Material



## Data Availability

The data that support the findings of this study are openly available on Zenodo at https://zenodo.org/records/20415624.

## Notes

Additional seam sampling and diabatization information, comparison of diabatization methods for PCET and PCEnT in triad **1**, definition of the phenol-pyridine dihedral angle, discussion of non-Condon effects and diabatic coupling gradients in triad **1**, seam sampling and reaction coordinate plots for PCET in triad **1** and PCEnT in triad **4** with the ωB97X-D functional, and confirmation of a conical intersection for triad **3** (PDF)
