# Supplementary material for: Comparing Nuclear Reaction Coordinates Driving Photoinduced Proton-Coupled Electron and Energy Transfer
Source: J Am Chem Soc. 2026 Oct 26;148(43):47216–27. doi: 10.1021/jacs.6c15580 (PMC13741824; doi:10.1021/jacs.6c15580)
Supplement: Supplementary file 1 [file ja6c15580_si_001.pdf]

Supporting Information
Comparing Nuclear Reaction Coordinates Driving Photoinduced Proton-Coupled Electron and Energy Transfer

Andreas Ghosh, Kai Cui, D. Vale Cofer-Shabica, Joseph E. Subotnik,* and Sharon Hammes-Schiffer*

Department of Chemistry, Princeton University, Princeton, NJ 08544, United States
E-mail: subotnik@princeton.edu, shs566@princeton.edu

Contents

S1 ADDITIONAL METHODOLOGICAL DETAILS **S3**
S1.1 Mathematical Details of Diabatization S3
S1.2 Diabatic Gradients S3
S1.3 E-SHAKE Trajectory Details S4

S2 ADDITIONAL RESULTS FOR TRIAD 1 **S5**
S2.1 Definition of Phenol–Pyridine Dihedral Angle S5
S2.2 Comparison of Diabatization Methods for PCET and PCEnT in Triad **1** S6
S2.3 Non-Condon Effects and Diabatic Electronic Coupling Gradients S7
S2.4 Proton Vibrational Wavefunction Overlap Integrals S8

S3 RESULTS WITH THE ωB97X-D FUNCTIONAL **S9**
S3.1 Seam Sampling Plots with the ωB97X-D Functional S9
S3.2 Reaction Coordinates with the ωB97X-D Functional S10

S4 CONFIRMATION OF A CI IN TRIAD 3 **S11**

S1 ADDITIONAL METHODOLOGICAL DETAILS

S1.1 Mathematical Details of Diabatization

For Boys diabatization, where we wish to produce diabatic states $|\Xi\rangle$ with localized charge, we can maximize the spread of the dipole operator:

$$f_{\text{Boys}}(\mathbf{U}) = \sum_{i,j=1}^{N_{\text{states}}} \|\langle\Xi_i|\hat{\mu}|\Xi_i\rangle - \langle\Xi_j|\hat{\mu}|\Xi_j\rangle\|^2 \tag{S1}$$

This diabatization technique works for charge transfer reactions because the donor and acceptor states have significantly different charge densities. In contrast, EnT reactions do not always involve charge rearrangement, but rather the spatial rearrangement of electronic excitations. Therefore, BoysOV diabatization separately maximizes the spread of the occupied (hole) and virtual (particle) parts of the dipole operator:

$$f_{\text{BoysOV}}(\mathbf{U}) = \sum_{i,j}^{N_{\text{states}}} \|\langle\Xi_i|\hat{\mu}^{\text{occ}}|\Xi_i\rangle - \langle\Xi_j|\hat{\mu}^{\text{occ}}|\Xi_j\rangle\|^2 + \|\langle\Xi_i|\hat{\mu}^{\text{vir}}|\Xi_i\rangle - \langle\Xi_j|\hat{\mu}^{\text{vir}}|\Xi_j\rangle\|^2 \tag{S2}$$

The appendix of the original BoysOV paper[1] shows the partitioning of the total dipole operator into its occupied and virtual parts.

In contrast to both Boys and BoysOV diabatization, which maximize the spread of the dipole operator (or its occupied and virtual components), Edmiston-Ruedenberg (ER) diabatization maximizes the self-interaction energy between diabats:[2]

$$f_{\text{ER}}(\mathbf{U}) = \sum_{i=1}^{N} \int d\mathbf{r}_1 \int d\mathbf{r}_2 \frac{\langle\Xi_i|\,\widehat{\rho}\,(\mathbf{r}_1)\,|\Xi_i\rangle\,\langle\Xi_i|\,\widehat{\rho}\,(\mathbf{r}_2)\,|\Xi_i\rangle}{|\mathbf{r}_1 - \mathbf{r}_2|} \tag{S3}$$

Here, the density operator is defined as

$$\widehat{\rho}(\mathbf{r}) = \sum_{i=1}^{\text{all electrons}} \delta\left(\mathbf{r} - \mathbf{r_i}\right) \tag{S4}$$

One advantage of ER diabatization is its applicability to both ET and EnT systems, although it is more computationally expensive than Boys and BoysOV diabatizations.

S1.2 Diabatic Gradients

Diabatic gradients are needed for E-SHAKE to satisfy the velocity constraint in eq 6 and to compute the reaction coordinate given in eq 1. We compute these gradients via the coupled-perturbed Hartree-Fock equations.[3] We speed up this method by invoking the strictly diabatic approximation, which assumes that the derivative coupling between diabatic states is exactly zero:

$$\langle\Xi_{\text{D}}|\nabla_{\mathbf{R}}|\Xi_{\text{A}}\rangle = 0 \tag{S5}$$

$$U_{AI}^{[\mathbf{R}]} = \sum_J U_{AI}\mathbf{d}_{JI} \tag{S6}$$

Here, U_{AI} is an element of the rotation matrix $\mathbf{U}$, $\mathbf{d}_{JI}$ is the derivative coupling between adiabats J and I (in eq 9 of the main text), and $U_{AI}^{[\mathbf{R}]}$ is an element of the nuclear gradient of the rotation matrix. The strictly diabatic approximation allows us to compute this nuclear gradient around an order of magnitude faster. The matrix gradient is needed to compute diabatic gradients because of the product rule.[4] Two important points should be noted here. First, the diabatic gradient is not exact, but the resulting error will not matter because we propagate along the adiabatic surfaces and always insist that the energy gap constraint in eq 5 is satisfied. Second, there are exact methods[5] to compute diabatic gradients for TDDFT states,[6] but they are slightly more expensive and therefore have not been included.

S1.3 E-SHAKE Trajectory Details

The E-SHAKE trajectories were not all propagated for the same amount of time because the computational time required for a single step of seam sampling differed for the systems studied. The total simulation times for each system are given in Table S1. The thermostat temperatures and timestep sizes used should not be interpreted as physically meaningful but rather as parameters tuned to sample the seam space more effectively. Because the thermostat was not very tight for certain trajectories, we labeled our results by the instantaneous temperatures instead.

Table S1: Total Number of Seam Sampling Steps and Corresponding Simulation Times for Each System Studied[a]

	Triad **1** LES–CSS	Triad **3** LES–CSS	Triad **1** LES–LEPT	Triad **4** LES–LEPT
Total Number of Seam Sampling Steps	4196	4129	2455	3807
Approximate Total Simulation Time (fs)	2029	1997	1188	1842

[a]The energies in the LES-LEPT seam sampling were more difficult to converge, leading to shorter simulation times due to high computational cost.

S2 ADDITIONAL RESULTS FOR TRIAD 1

S2.1 Definition of Phenol–Pyridine Dihedral Angle

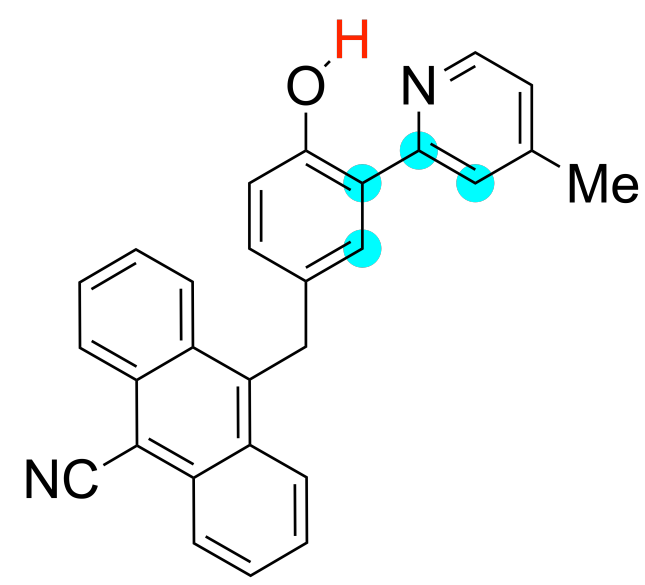

Figure S1: Structure of triad **1** with the four atoms used to define the phenol–pyridine dihedral angle highlighted in light blue. At the DMECP geometry for PCET, this angle is 14.1°, whereas it is 0.3° at the DMECP geometry for PCEnT.

S2.2 Comparison of Diabatization Methods for PCET and PCEnT in Triad 1

We evaluated the impact of the diabatization scheme on our results by calculating the diabatic energy gap and the diabatic electronic coupling with the ER diabatization scheme for ten representative geometries of triad **1** . These geometries were obtained from the diabatic seam sampling performed for PCET and PCEnT using Boys or BoysOV diabatization, respectively. A comparison of these quantities computed with ER diabatization versus Boys or BoysOV diabatization is provided in Table S2. The diabatic energy gaps are all very small, as expected in the diabatic seam, where the energy gap threshold used for E-SHAKE was 4×10^{-5} a.u. The diabatic electronic couplings are the same to within the three significant figures shown.

Table S2: Comparison of Diabatization Methods for PCET and PCEnT in Triad **1**[a]

	Diabatic Energy Gap (a.u.)	Diabatic Electronic Coupling (a.u.)
PCET Geometry 1 (Boys)	1.01×10^{-6}	5.03×10^{-5}
PCET Geometry 1 (ER)	1.09×10^{-6}	5.03×10^{-5}
PCET Geometry 2 (Boys)	1.07×10^{-5}	8.03×10^{-5}
PCET Geometry 2 (ER)	1.08×10^{-5}	8.03×10^{-5}
PCET Geometry 3 (Boys)	6.78×10^{-7}	1.22×10^{-4}
PCET Geometry 3 (ER)	8.22×10^{-7}	1.22×10^{-4}
PCET Geometry 4 (Boys)	1.31×10^{-6}	9.36×10^{-4}
PCET Geometry 4 (ER)	3.55×10^{-7}	9.36×10^{-4}
PCET Geometry 5 (Boys)	1.36×10^{-5}	1.13×10^{-3}
PCET Geometry 5 (ER)	1.57×10^{-5}	1.13×10^{-3}
PCEnT Geometry 1 (BoysOV)	8.82×10^{-6}	1.09×10^{-3}
PCET Geometry 1 (ER)	5.40×10^{-5}	1.09×10^{-3}
PCEnT Geometry 2 (BoysOV)	3.44×10^{-6}	1.14×10^{-3}
PCEnT Geometry 2 (ER)	5.07×10^{-5}	1.14×10^{-3}
PCEnT Geometry 3 (BoysOV)	7.29×10^{-6}	1.22×10^{-3}
PCEnT Geometry 3 (ER)	5.94×10^{-5}	1.22×10^{-3}
PCEnT Geometry 4 (BoysOV)	1.77×10^{-6}	1.68×10^{-3}
PCEnT Geometry 4 (ER)	8.32×10^{-5}	1.68×10^{-3}
PCEnT Geometry 5 (BoysOV)	2.11×10^{-5}	1.56×10^{-3}
PCEnT Geometry 5 (ER)	5.42×10^{-5}	1.56×10^{-3}

[a]Geometries were obtained from diabatic seam sampling using Boys or BoysOV diabatization for PCET and PCEnT, respectively. The absolute values of the diabatic electronic couplings are reported. Note that the diabatic electronic couplings are the same to within the three significant figures shown but differ in the fifth or sixth significant figure.

S2.3 Non-Condon Effects and Diabatic Electronic Coupling Gradients

We begin our analysis of non-Condon effects by examining the nuclear gradient of the diabatic electronic coupling, $\nabla_{\mathbf{R}} H_{\mathrm{DA}}$, at the DMECP geometries for PCET and PCEnT in triad **1**, shown on the left side of Figure S2 with dark purple arrows. In the PCET system, significant contributions to this gradient arise from the out-of-plane motions of the phenol and anthracene rings, whereas in the PCEnT system, the significant contributions arise from in-plane stretching of the pyridine nitrogen atom and its neighboring carbon atoms. The ratio of the magnitudes of this vector for PCET to PCEnT is ≈ 2, indicating that the PCET diabatic electronic coupling depends more strongly on nuclear motion in the vicinity of the DMECP. This stronger dependence contributes to the larger spread in diabatic electronic couplings for PCET close to the DMECP, as discussed in the main text.

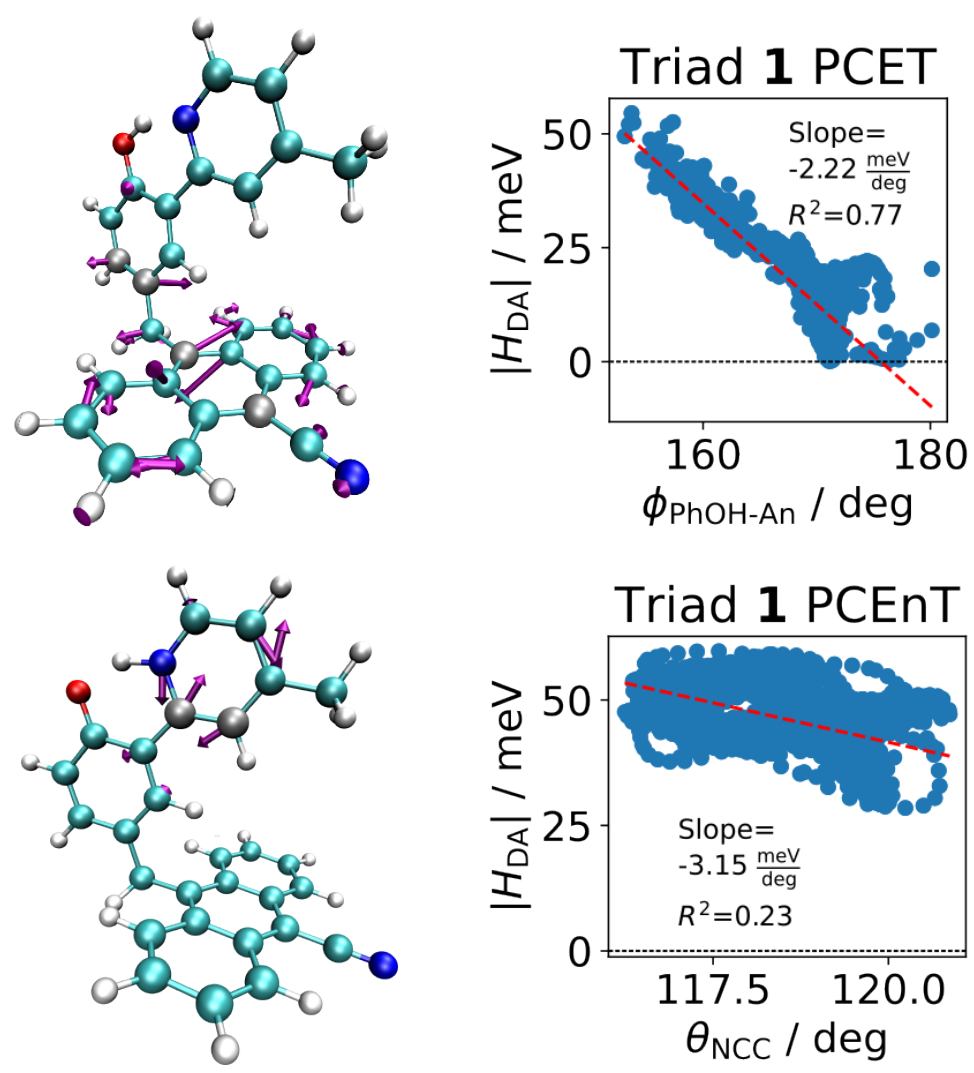

Figure S2: Left: nuclear gradients of the diabatic electronic coupling resolved onto each atom for PCET (top) and PCEnT (bottom). The dihedral angle $\phi_{\text{PhOH-An}}$ for PCET and the angle θ_{NCC} for PCEnT are defined by the carbon atoms in gray, as well as the nitrogen for PCEnT. Right: plots of the diabatic electronic coupling versus the angles $\phi_{\text{PhOH-An}}$ and θ_{NCC} for geometries in the seam space up to 500 meV above the DMECP. When fit to straight lines, the PCET data has a stronger correlation, with an R^2 value of 0.77, compared to 0.23 for PCEnT.

To better understand how these nuclear motions govern the diabatic electronic coupling, for each system we plot the absolute value of the coupling (for geometries in the seam space sampled up to 500 meV) versus a nuclear coordinate related to the coupling. For the PCET system, where the out-of-plane motions of the phenol and anthracene rings strongly change the diabatic electronic coupling, we plot the absolute value of the coupling versus the phenol–anthracene dihedral angle $\phi_{\text{PhOH-An}}$. This definition of the dihedral angle is nonstandard, but it captures important non-Condon effects for the PCET system. For the PCEnT system, where the in-plane stretching

motions of the pyridine ring strongly change the diabatic electronic coupling, we plot the absolute value of the coupling versus the pyridine N–C–C bond angle θ_{NCC}. Admittedly, these coordinates do not completely capture all non-Condon effects within the diabatic seam spaces of these systems, but this simplification allows us to more easily understand how the diabatic electronic coupling changes with nuclear geometry. The definitions of these coordinates and the plots of their relation to the diabatic electronic coupling are shown in Figure S2. By fitting our data to a straight line, we find that the PCET diabatic electronic coupling has a stronger correlation with $\phi_{\mathrm{PhOH\text{-}An}}$ than the PCEnT coupling has with θ_{NCC}, with R^2 values of 0.77 and 0.23, respectively. This analysis shows differences between the dependence of the diabatic electronic coupling on nuclear coordinates for PCET and PCEnT.

S2.4 Proton Vibrational Wavefunction Overlap Integrals

Table S3: Proton Vibrational Wavefunction Overlap Integrals Associated with the First Two Reactant and Product Vibronic States for PCET and PCEnT in Triad **1**

	$\lvert S_{00}^{\mathrm{H}}\rvert^2$	$\lvert S_{00}^{\mathrm{D}}\rvert^2$	$\lvert S_{01}^{\mathrm{H}}\rvert^2$	$\lvert S_{01}^{\mathrm{D}}\rvert^2$	$\lvert S_{10}^{\mathrm{H}}\rvert^2$	$\lvert S_{10}^{\mathrm{D}}\rvert^2$	$\lvert S_{11}^{\mathrm{H}}\rvert^2$	$\lvert S_{11}^{\mathrm{D}}\rvert^2$
Triad **1** PCET	0.179	0.032	0.653	0.741	0.251	0.041	0.023	0.164
Triad **1** PCEnT	0.012	0.006	0.138	0.011	0.381	0.059	0.305	0.164

S3 RESULTS WITH THE ωB97X-D FUNCTIONAL

S3.1 Seam Sampling Plots with the ωB97X-D Functional

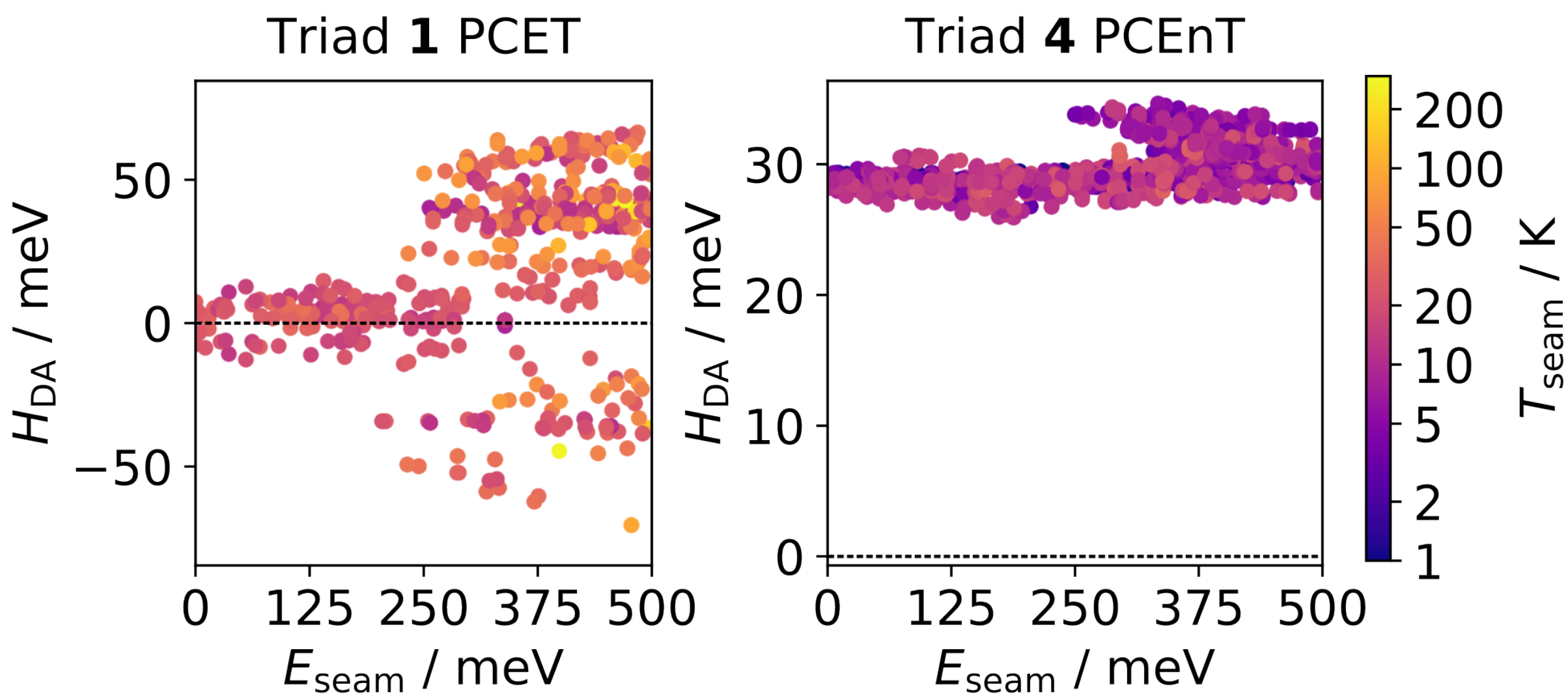

Figure S3: Diabatic electronic couplings between the LES and CSS for PCET in triad **1** or between the LES and LEPT state for PCEnT in triad **4** versus the energy of its lower adiabatic surface relative to its DMECP, calculated with the ωB97X-D functional. As in the main text, these diabatic seams appear to violate the Condon approximation, and we observe an energetically relevant intersection of adiabatic surfaces in the diabatic seam for PCET but not for PCEnT.

Diabatic seam sampling results obtained with the ωB97X-D functional are shown in Figure S3. Despite the significantly smaller amount of sampling, this plot is qualitatively similar to the analogous plots in Figure 3 of the main text. The Condon approximation is violated for both the PCET and PCEnT systems, but the variation in the diabatic electronic couplings is much smaller for PCEnT. We also observe a sign change in the diabatic coupling for PCET and strictly positive diabatic couplings for PCEnT, as found with the CAM-B3LYP functional in the main text. The maximum magnitude of the diabatic coupling computed with the ωB97X-D functional is larger than that computed with the CAM-B3LYP functional, but such quantitative differences are expected with different functionals.

S3.2 Reaction Coordinates with the ωB97X-D Functional

We now compare the reaction coordinates obtained for PCET in triad **1** and PCEnT in triad **4** obtained with the CAM-B3LYP and ωB97X-D functionals. The reaction coordinates are qualitatively similar between the two functionals, indicating that the choice of functional does not significantly impact our analysis.

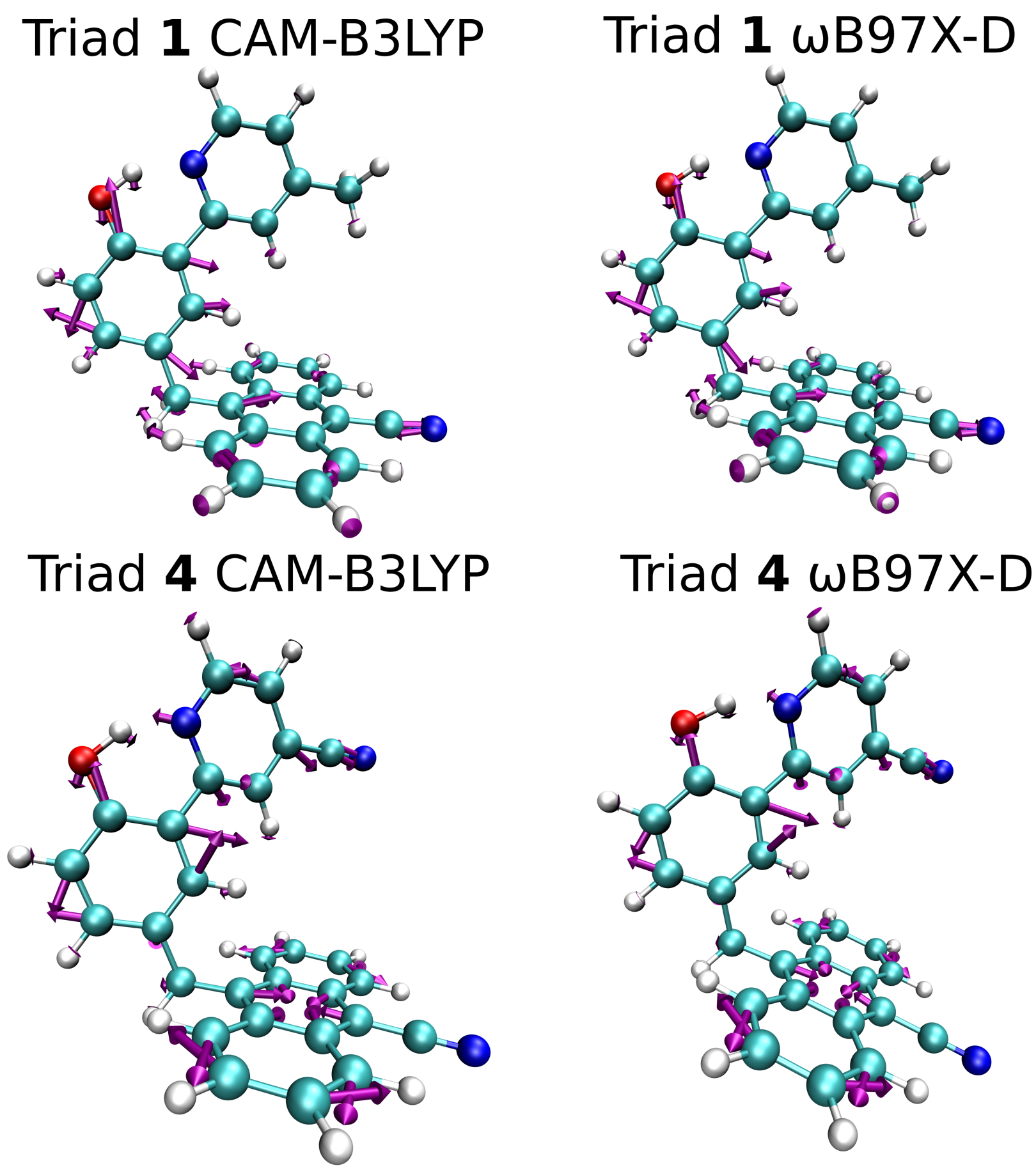

Figure S4: The DMECP geometries and reaction coordinates for PCET in triad **1** and PCEnT in triad **4** calculated using the CAM-B3LYP functional and the ωB97X-D functional with the reaction coordinate vectors plotted on each atom in purple. The reaction coordinate vectors are qualitatively the same, showing that the analysis and conclusions in the paper are not sensitive to differences between these two functionals.

S4 CONFIRMATION OF A CI IN TRIAD 3

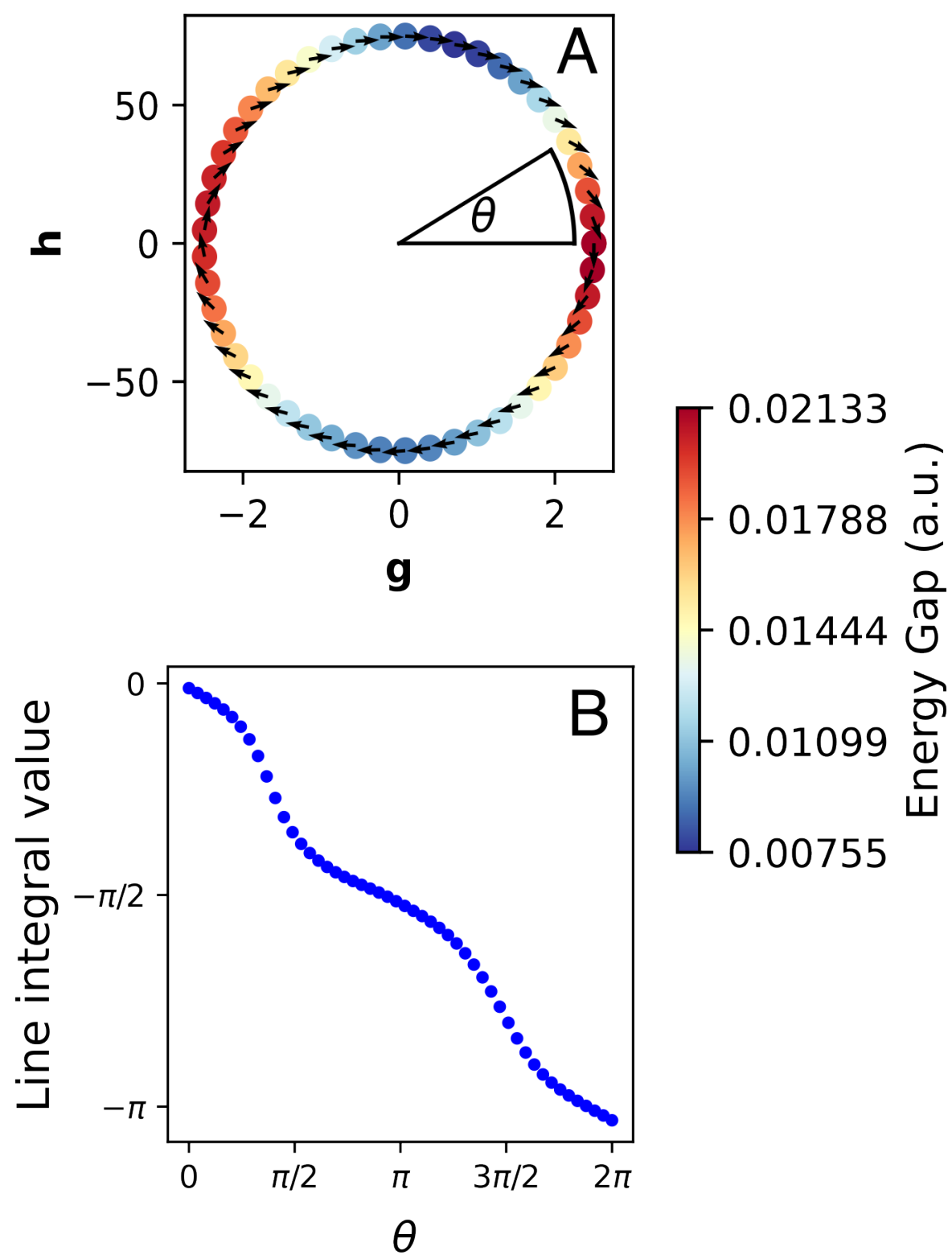

Figure S5: (A) Depiction of the triad **3** branching plane with the **g** and **h** vectors as axes. Each point is a single-point TDDFT calculation along the path given by eq 11 and is colored by the magnitude of the energy gap between the S_1 and S_2 adiabatic surfaces. The arrow on each point is the projection of the derivative coupling vector in the branching plane. A nonzero energy gap opens up in both the **g** and **h** directions, and the derivative coupling varies smoothly, indicating a suitable path for the line integral. The angle of integration θ for the line integral is also shown. (B) The value of the line integral evaluated from $\theta = 0$ along the positive x-axis to the angle θ measured relative to the x-axis. The integral evaluates to -1.03π for triad **3**, proving that the intersection of adiabats is indeed conical.

References

(1) Subotnik, J. E.; Vura-Weis, J.; Sodt, A. J.; Ratner, M. A. Predicting Accurate Electronic Excitation Transfer Rates via Marcus Theory with Boys or Edmiston-Ruedenberg Localized Diabatization. *J. Phys. Chem. A* **2010**, *114*, 8665–8675.

(2) Alguire, E.; Subotnik, J. E. Optimal diabatic states based on solvation parameters. *J. Chem. Phys.* **2012**, *137*, 194108.

(3) Fatehi, S.; Alguire, E.; Subotnik, J. E. Derivative couplings and analytic gradients for diabatic states, with an implementation for Boys-localized configuration-interaction singles. *J. Chem. Phys.* **2013**, *139*, 124112.

(4) Alguire, E. C.; Fatehi, S.; Shao, Y.; Subotnik, J. E. Analysis of Localized Diabatic States beyond the Condon Approximation for Excitation Energy Transfer Processes. *J. Phys. Chem. A* **2014**, *118*, 11891–11900.

(5) Paz, A. S. P.; Glover, W. J. Efficient analytical gradients of property-based diabatic states: Geometry optimizations for localized holes. *J. Chem. Phys.* **2023**, *158*, 204107.

(6) Athavale, V.; Subotnik, J. E.; Qiu, T. Evaluating the gradients of localized diabatic state energies and couplings at minimum cost. *J. Chem. Phys.* **2025**, *163*, 024116.
